# Supplementary material for: Concurrent gliomas in patients with multiple sclerosis
Source: Commun Med (Lond). 2023 Dec 18;3:186. doi: 10.1038/s43856-023-00381-y (PMC10728097; doi:10.1038/s43856-023-00381-y)
Supplement: Supplementary file 1 — Description of Additional Supplementary Files [file 43856_2023_381_MOESM1_ESM.pdf]

Description of Additional Supplementary Files

File Name: Supplementary Data 1

Description: Table summarizing glioma characteristics of all patients included in this study (n=49).

*Excluded due to failure of predefined inclusion criteria; **Censored

B = Biopsy; CR = Complete resection; CT = Chemotherapy; PR = Partial resection; RT = Radiotherapy

File Name: Supplementary Data 2

Description: Source code applied to analyze Illumina 450k methylation array data.
