# Supplementary material for: Concurrent gliomas in patients with multiple sclerosis
Source: Commun Med (Lond). 2023 Dec 18;3:186. doi: 10.1038/s43856-023-00381-y (PMC10728097; doi:10.1038/s43856-023-00381-y)
Supplement: Supplementary file 2 — Supplementary Material [file 43856_2023_381_MOESM2_ESM.pdf]

Supplementary Material

Concurrent gliomas in patients with multiple sclerosis

Katharina Sahm, Tobias Kessler, Philipp Eisele, Miriam Ratliff, Elena Sperk, Laila König, Michael O. Breckwoldt, Corinna Seliger, Iris Mildenberger, Daniel Schrimpf, Christel Herold-Mende, Pia S. Zeiner, Ghazaleh Tabatabai, Sven G. Meuth, David Capper, Martin Bendszus, Andreas von Deimling, Wolfgang Wick, Felix Sahm, and Michael Platten

Supplementary Figure 1

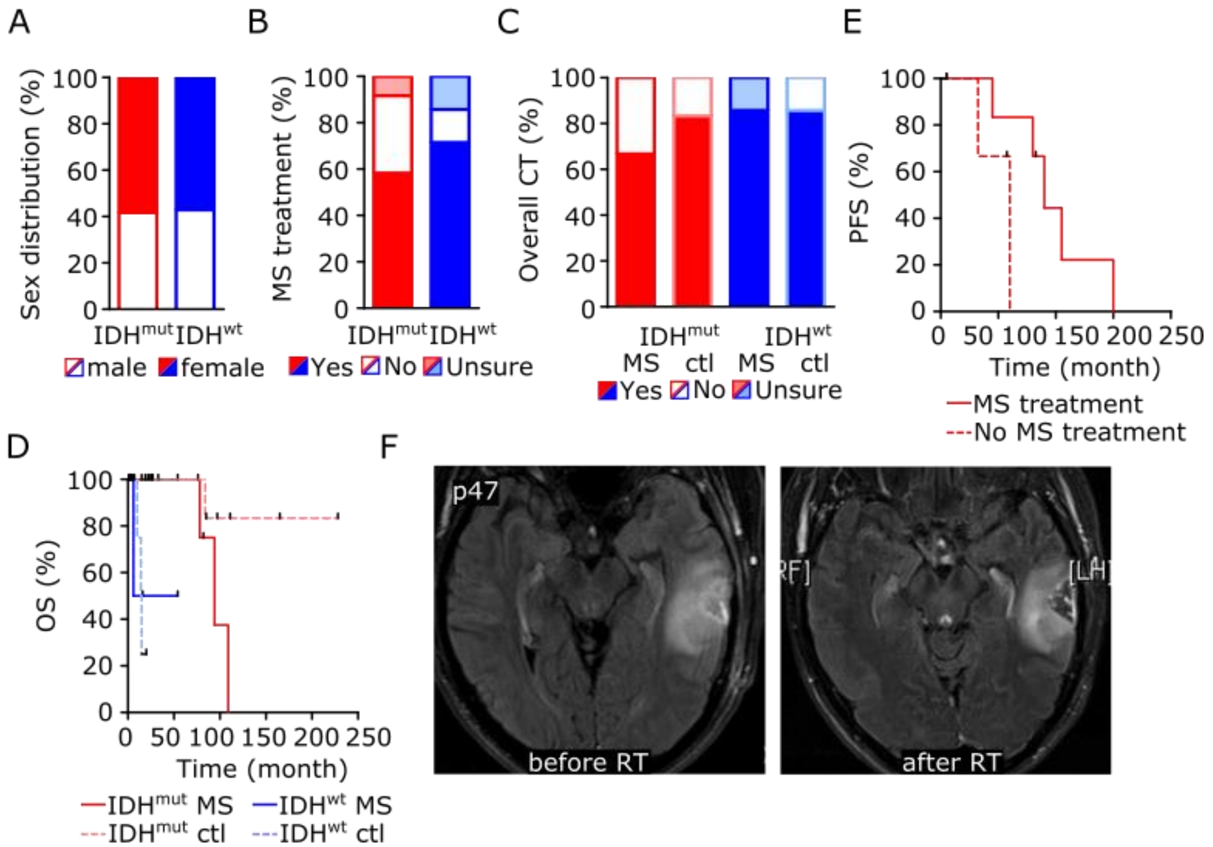

Clinical and radiological characteristics of 19 patients with multiple sclerosis and concurrent glioma.

A Sex distribution of patient and control cohorts.

B Percentage of patients with and without disease-modifying treatment during the course of multiple sclerosis (MS) depending on tumoral isocitratdehydrogenase-1 (IDH) mutation status.

C Percentage of patients with and without chemotherapy (CT) treatment during the course of brain tumor disease depending on concurrent MS diagnosis and tumoral IDH mutation status.

D Kaplan–Meier analysis of overall survival (OS) after primary tumor treatment depending on IDH status and concurrent multiple sclerosis diagnosis (IDH-mut MS n=12; IDH-mut ctl n=12; IDH-wt MS n=7; IDH-wt ctl n=7).

E Kaplan–Meier analysis of progression-free survival (PFS) of patients with IDH mutant astrocytoma and concurrent multiple sclerosis after primary tumor treatment depending on immunomodulatory therapy (MS treatment n=7; No MS treatment n=4).

F MRI with axial FLAIR images of patient p47 with IDH-mutant astrocytoma showing the tumoral lesion before and after RT with stable disease according to RANO criteria.

Supplementary Figure 2

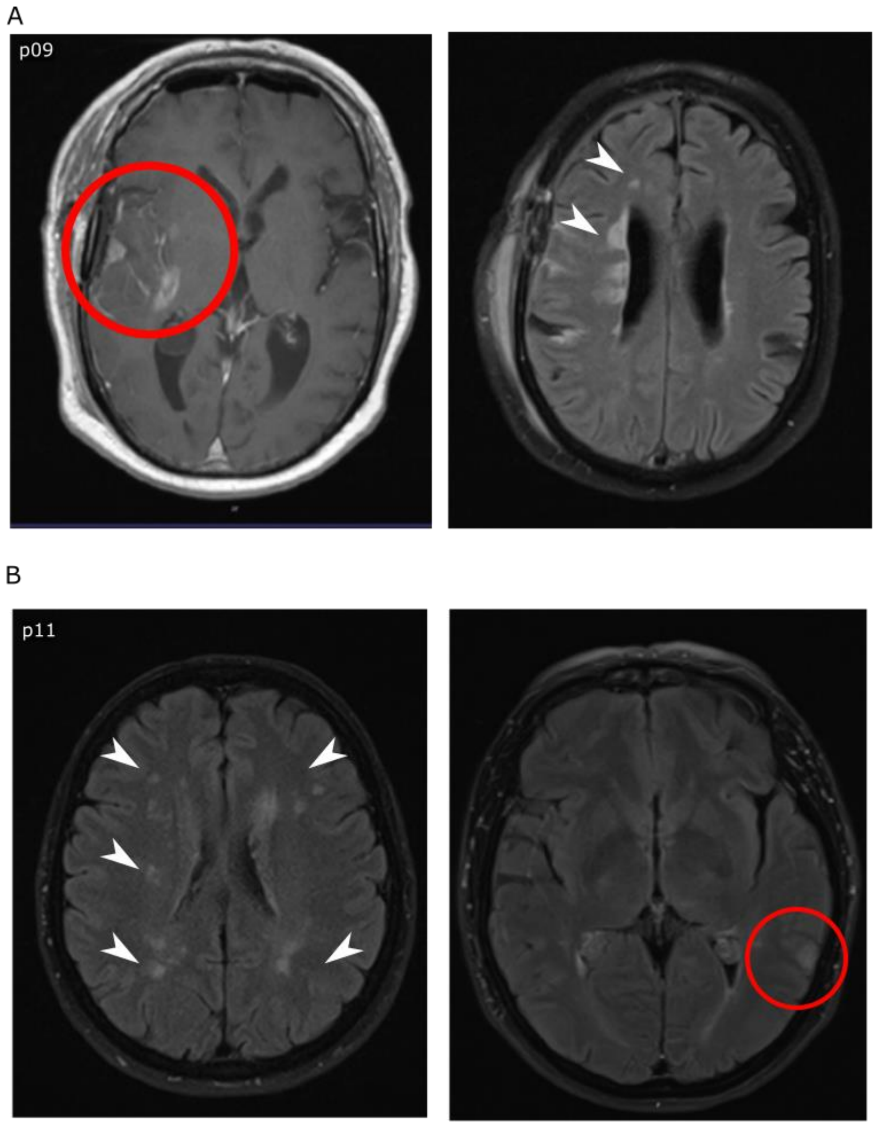

A MRI with axial contrast-enhanced T1 (left) and FLAIR images (right) of a patient with known multiple sclerosis at the time of the initial diagnosis of an isocitratdehydrogenase-1 (IDH)-wildtype glioblastoma (p09)

B MRI with axial FLAIR images of a patient with known multiple sclerosis at the time of the initial diagnosis of an IDH-wildtype glioblastoma (p11)

Arrows: multiple sclerosis lesions; Red circle: Glioblastoma

Supplementary Figure 3

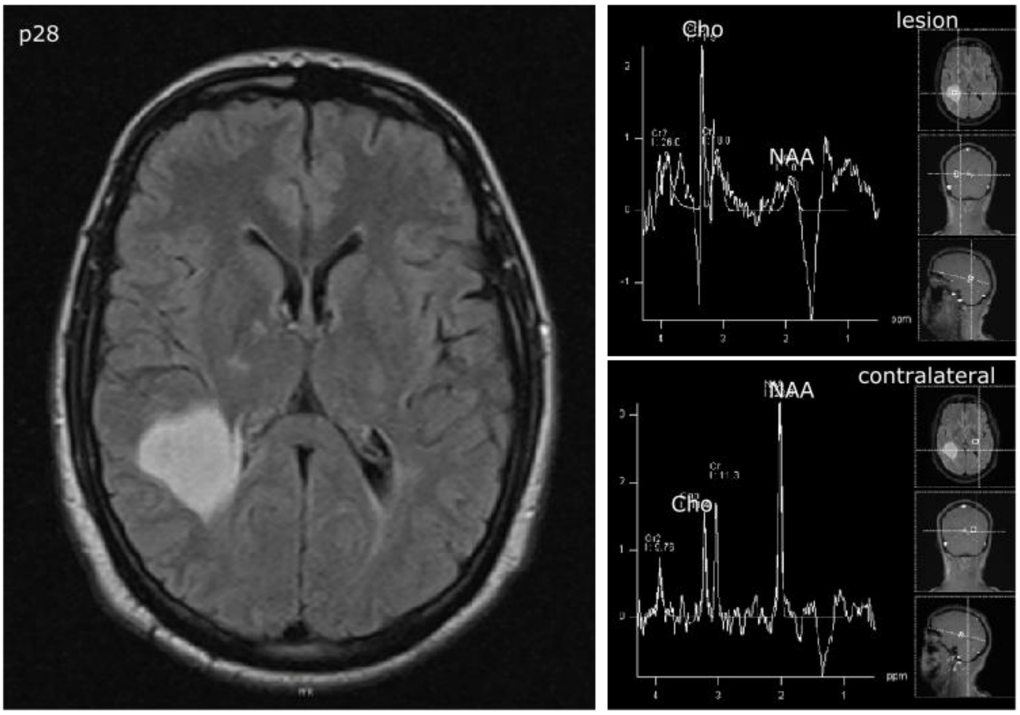

MRI spectroscopy

MRI scan of multiple sclerosis patient p28 with axial FLAIR images and spectroscopy demonstrating choline-increase and N-acetyl-aspartate decrease in an isocitratdehydrogenase-1 (IDH)-mutant astrocytoma WHO grade 2.

Supplementary Figure 4

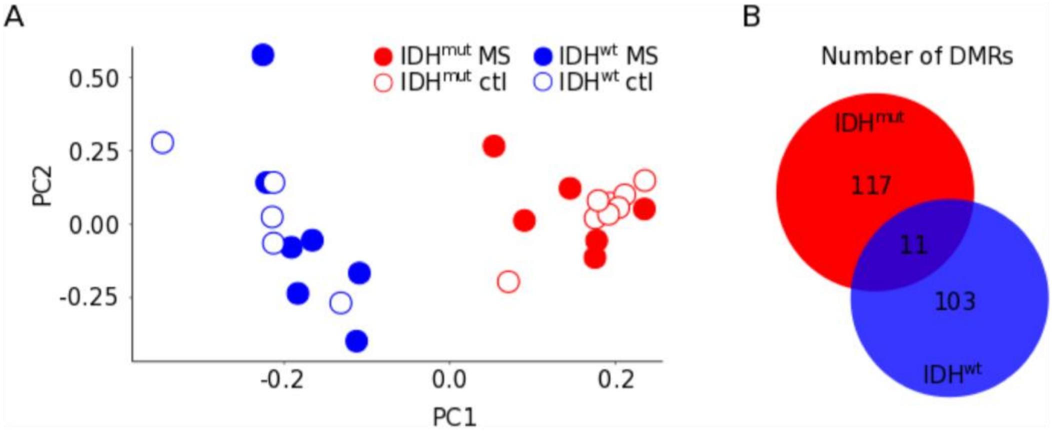

Genome-wide DNA methylation changes of gliomas in patients with concurrent multiple sclerosis

A Principal component analysis.

B Schematic representation of numbers of differentially methylated regions (DMRs) in gliomas compared between patients with concurrent multiple sclerosis and control patients without multiple sclerosis depending on tumoral isocitratdehydrogenase-1 (IDH) mutation status.

Supplementary Table 1 Characteristics of patients with glioma and concurrent multiple sclerosis (n=26)

Characteristic	Number of patients (%)
Male	10 (38%)
Median age at tumor diagnosis (y)	42 (15–67)
Tumor histology	
Astrocytoma	26 (100%)
Oligodendroglioma	0 (0%)
Tumor WHO grade	
1	3 (12%)
2	14 (54%)
3	2 (8%)
4	7 (27%)
IDH1 mutation status	
Wildtype	8 (31%)
Mutant	12 (46%)
Unsure	6 (23%)
MGMT promotor status	
Hypermethylated	13 (50%)
Hypomethylated	4 (15%)
Unsure	9 (35%)
Tumor location	
Frontal	7 (27%)
Temporal	12 (44%)
Parietal	1 (4%)
Occipital	4 (15%)
Brainstem	1 (4%)
Cerebellar	1 (4%)
Surgery	
Biopsy	14 (54%)
Partial resection	2 (8%)
Complete resection	10 (38%)
Overall tumor-specific therapy	
Radiotherapy	19 (73%)
Chemotherapy	18 (69%)
Median age at MS diagnosis (y)	33 (16–61)
MS symptom course	
Relapsing remitting MS	19 (73%)
Primary progressive MS	0 (0%)
Secondary progressive MS	4 (15%)
Inflammatory lesion location	
Optic neuritis	7 (27%)
Cerebral	26 (100%)
Spinal	6 (23%)
Overall immunomodulatory therapy	
Yes	15 (58%)
No	5 (19%)
Unsure	6 (23%)

Supplementary Table 2 Glioma risk in patients with multiple sclerosis

Study cohort	Total number of patients	Number of patients with MS (%)	
Glioma Heidelberg	2809	12 (0.4)	**Odds ratio (95% CI)**
Glioma CPRD*	3112	9 (0.3)	1.48 (0.62 – 3.52)
Control CPRD*	31120	85 (0.3)	1.57 (0.86 – 2.87)

Glioma risk among MS patients of the Glioma Heidelberg cohort was compared to risks of Glioma CPRD and Control CPRD cohorts previously published (*(Anssar *et al.*, 2020); 1:10 matched case-control study within the CPRD (Clinical Practice Research Datalink)).

Supplementary Table 3 Characteristics of patients with IDH1-wildtype glioblastoma WHO grade 4

**Characteristic**	**Number of MS patients (%) n=7 (100%)**	**Number of ctl patients (%) n=7 (100%)**
Male	3 (43%)	4 (57%)
Median age at tumor diagnosis (y)	59 (42–67)	59 (41–71)
Glioblastoma manifestation		
New symptoms	6 (86%)	6 (86%)
MRI incidental finding	0 (0%)	1 (14%)
Unsure	1 (14%)	0 (0%)
Tumor location		
Frontal	2 (29%)	2 (29%)
Temporal	2 (29%)	1 (14%)
Parietal	0 (0%)	2 (29%)
Occipital	3 (43%)	2 (29%)
Brainstem	0 (0%)	0 (0%)
Cerebellar	0 (0%)	0 (0%)
Surgery		
Biopsy	2 (29%)	1 (14%)
Partial resection	1 (14%)	4 (57%)
Complete resection	4 (57%)	2 (29%)
MGMT promotor status		
Hypermethylated	1 (14%)	2 (29%)
Hypomethylated	4 (57%)	4 (57%)
Unsure	2 (29%)	1 (14%)
1st treatment		
Best supportive care	0 (0%)	1 (14%)
Radiotherapy only	1 (14%)	0 (0%)
Chemotherapy only	1 (14%)	0 (0%)
Radiochemotherapy	5 (71%)	6 (86%)
Median PFS (month)	2.5	8

Supplementary Table 4 Characteristics of patients with IDH1-mutant astrocytoma WHO grade 2

Characteristic	Number of MS patients (%) n=12 (100%)	Number of ctl patients (%) n=12 (100%)
Male	5 (42%)	8 (67%)
Median age at tumor diagnosis (y)	32.5 (21–48)	31.5 (22–49)
Tumor manifestation		
Symptomatic	4 (33%)	12 (100%)
MRI incidental finding	7 (58%)	0 (0%)
Unsure	1 (8%)	0 (0%)
Tumor location		
Frontal	4 (33%)	9 (75%)
Temporal	7 (58%)	3 (25%)
Parietal	0 (0%)	0 (0%)
Occipital	1 (8%)	0 (0%)
Brainstem	0 (0%)	0 (0%)
Cerebellar	0 (0%)	0 (0%)
Surgery		
Biopsy	6 (50%)	3 (25%)
Partial resection	1 (8%)	3 (25%)
Complete resection	5 (42%)	6 (50%)
1st treatment		
Watch + wait	7 (58%)	6 (50%)
Radiotherapy	3 (25%)	3 (25%)
Chemotherapy	2 (17%)	5 (42%)
Unsure	1 (8%)	0 (0%)
Median PFS (month)	32	64

Supplementary Table 5: Multiple sclerosis course

	IDH-mutant, WHO grade 2	IDH-wildtype, WHO grade 4
Mean age at MS manifestation (y)	31 (16–47)	unsure
Mean age at MS diagnosis (y)	31 (16–48)	40 (30–61)
Disease sequence		
Glioma first	4 (33%)	0 (0%)
Simultaneous	6 (50%)	0 (0%)
MS first	1 (8%)	7 (100%)
median time between MS and tumor diagnosis (y)	0 (-7–13)	13 (1–34)
Cerebral inflammatory lesions	12 (100%)	7 (100%)
Overall immunomodulatory therapy		
Yes	7 (58%)	3 (43%)
No	4 (33%)	1 (14%)
Unsure	1 (8%)	3 (43%)
Overall RT	10 (83%)	5 (71%)
MS progression in the 12 months preceding RT	0 (0%)	0 (0%)
MS progression in the 12 months after RT	5 (42%)*, **	1 (20%)*
Overall CT	8 (67%)	6 (86%)
MS progression in the 12 months preceding CT	0 (0%)	0 (0%)
MS progression in the 12 months after CT	1 (12%)*	1 (17%)*

* combined RCT, ** three (60%) with immunomodulatory treatment

Supplementary Table 6 Common differentially methylated regions (DMR)

Genetic region	Annotated gene	Immune-related function
3q24	ZIC4	
4q23	EIF4E	Regulation of immune function
5q31.3	TMCO6	Interleukin region
5q31.3	PCDHB3	Interleukin region
5q35.1	DUSP1	Autoimmune disease risk
5p15.33	C5orf38	
6p22.1	RNF39	HLA region
6p21.32	PRRT1	HLA region
10p12.1	MKX	
12p13.1	GPR19	
18p11.21	CIDEA	
