# Supplementary material for: Concurrent gliomas in patients with multiple sclerosis
Source: Commun Med (Lond). 2023 Dec 18;3:186. doi: 10.1038/s43856-023-00381-y (PMC10728097; doi:10.1038/s43856-023-00381-y)
Supplement: Supplementary file 4 — Reporting Summary [file 43856_2023_381_MOESM4_ESM.pdf]

nature portfolio

Corresponding author(s): Katharina Sahm & Michael Platten

Last updated by author(s): 19-SEP-2023

Reporting Summary

Nature Portfolio wishes to improve the reproducibility of the work that we publish. This form provides structure for consistency and transparency in reporting. For further information on Nature Portfolio policies, see our Editorial Policies and the Editorial Policy Checklist.

Statistics

For all statistical analyses, confirm that the following items are present in the figure legend, table legend, main text, or Methods section.

n/a	Confirmed	
☐	☒	The exact sample size (*n*) for each experimental group/condition, given as a discrete number and unit of measurement
☒	☐	A statement on whether measurements were taken from distinct samples or whether the same sample was measured repeatedly
☐	☒	The statistical test(s) used AND whether they are one- or two-sided<br>*Only common tests should be described solely by name; describe more complex techniques in the Methods section.*
☒	☐	A description of all covariates tested
☒	☐	A description of any assumptions or corrections, such as tests of normality and adjustment for multiple comparisons
☐	☒	A full description of the statistical parameters including central tendency (e.g. means) or other basic estimates (e.g. regression coefficient) AND variation (e.g. standard deviation) or associated estimates of uncertainty (e.g. confidence intervals)
☐	☒	For null hypothesis testing, the test statistic (e.g. *F*, *t*, *r*) with confidence intervals, effect sizes, degrees of freedom and *P* value noted<br>*Give P values as exact values whenever suitable.*
☒	☐	For Bayesian analysis, information on the choice of priors and Markov chain Monte Carlo settings
☒	☐	For hierarchical and complex designs, identification of the appropriate level for tests and full reporting of outcomes
☒	☐	Estimates of effect sizes (e.g. Cohen's *d*, Pearson's *r*), indicating how they were calculated

Our web collection on statistics for biologists contains articles on many of the points above.

Software and code

Policy information about availability of computer code

Data collection	Data were extracted from local databases, no custom software was used.
Data analysis	Bioinformatic analysis of Illumina 450k methylation array data was performed with customized scripts adapted from the R (www.r-project.org) based library "ChAMP" (version: 2.10.116) . Source code is described in Supplementary Data File 2. Circos plots showing DMR distribution were constructed with the package "OmicCircos" (version: 1.26.0). Clinical data were analyzed using GraphPad Prism Version 8.4.2.

For manuscripts utilizing custom algorithms or software that are central to the research but not yet described in published literature, software must be made available to editors and reviewers. We strongly encourage code deposition in a community repository (e.g. GitHub). See the Nature Portfolio guidelines for submitting code & software for further information.

Data

Policy information about availability of data

All manuscripts must include a data availability statement. This statement should provide the following information, where applicable:

- Accession codes, unique identifiers, or web links for publicly available datasets
- A description of any restrictions on data availability
- For clinical datasets or third party data, please ensure that the statement adheres to our policy

Source data for Figures 2-4 can be found in Supplementary Data File 1 and Supplementary Tables 1-5. Methylation data are deposited at the Gene Expression Omnibus data repository (accession number GSE243465).

March 2021

Human research participants

Policy information about studies involving human research participants and Sex and Gender in Research.

Reporting on sex and gender	Due to the rare coincidence of multiple sclerosis and diffuse glioma resulting in a small sample size of this retrospective study, sex- or gender-based analyses were not appropriate.
Population characteristics	The study population includes 26 patients diagnosed with both multiple sclerosis and diffuse glioma (12 patients with IDH-mutant astrocytoma WHO grade 2 and 7 patients with IDH-wildtype glioblastoma WHO grade 4, respectively). Median age at tumor diagnosis was 32.5 years for IDH-mutant astrocytomas and 59 years for glioblastomas.
Recruitment	In this multicenter, retrospective analysis, 30 brain tumor tissue samples diagnosed between 01 January 2001 and 31 December 2017 and clinical and radiographical data of patients with reported multiple sclerosis were assessed for eligability. Control cases with isocitrate dehydrogenase (IDH)-mutant 1p19q-intact astrocytoma WHO grade II or IDH-wildtype glioblastoma WHO grade IV were collected and matched for tumor histology and the known prognostic markers IDH- and O6-methylguanine-DNA methyltransferase (MGMT)-status, age at diagnosis, and Karnofsky-Index.
Ethics oversight	Tissue sample collection and data collection and use were performed following local ethics regulations and approved by the local ethics committee.

Note that full information on the approval of the study protocol must also be provided in the manuscript.

Field-specific reporting

Please select the one below that is the best fit for your research. If you are not sure, read the appropriate sections before making your selection.

☒ Life sciences ☐ Behavioural & social sciences ☐ Ecological, evolutionary & environmental sciences

For a reference copy of the document with all sections, see nature.com/documents/nr-reporting-summary-flat.pdf

Life sciences study design

All studies must disclose on these points even when the disclosure is negative.

Sample size	The rare coincidence of multiple sclerosis and diffuse glioma resulted in a study population of 26 patients. Small sample and subgroup sizes have been discussed as potential limitation of the presented study.
Data exclusions	Data from four cases were excluded due to tumor histology other than glioma or insufficient proof of multiple sclerosis diagnosis according to the revised McDonald criteria,
Replication	n.a.
Randomization	n.a.
Blinding	n.a.

Reporting for specific materials, systems and methods

We require information from authors about some types of materials, experimental systems and methods used in many studies. Here, indicate whether each material, system or method listed is relevant to your study. If you are not sure if a list item applies to your research, read the appropriate section before selecting a response.

Materials & experimental systems

n/a	Involved in the study	
☒	☐	Antibodies
☒	☐	Eukaryotic cell lines
☒	☐	Palaeontology and archaeology
☒	☐	Animals and other organisms
☒	☐	Clinical data
☒	☐	Dual use research of concern

Methods

n/a	Involved in the study	
☒	☐	ChIP-seq
☒	☐	Flow cytometry
☒	☐	MRI-based neuroimaging
