# Supplementary material for: Concurrent gliomas in patients with multiple sclerosis
Source: Commun Med (Lond). 2023 Dec 18;3:186. doi: 10.1038/s43856-023-00381-y (PMC10728097; doi:10.1038/s43856-023-00381-y)
Supplement: Supplementary file 6 — Supplementary Data 2 [file 43856_2023_381_MOESM6_ESM.pdf]

Figure 6

The figure 6 was created with the following packages and settings.

```
library(ggplot2)
library(png)
library(grid)
library(plyr)
library(amap)
```

Preprocessing

Loading the methylation data

```
beta_values.GPL13534 <- read.table('GPL13534_matrix_processed.txt', sep='\t', header=TRUE)
beta_values.GPL13534 <- beta_values.GPL13534[,!grepl('Detection',
                                                     colnames(beta_values.GPL13534))]

beta_values.GPL21145 <- read.table('GPL21145_matrix_processed.txt',sep='\t', header=TRUE)
beta_values.GPL21145 <- beta_values.GPL21145[,!grepl('Detection',
                                                     colnames(beta_values.GPL21145))]

betas.tmp <- cbind(beta_values.GPL13534, beta_values.GPL21145[,-1])

betas <- data.matrix(betas.tmp[,-1])
rownames(betas) <- betas.tmp$ID_REF
colnames(betas) <- gsub('X','', colnames(betas))
```

Loading the annotation data

```
annotation_data <- read.table('annotation.txt', sep='\t', header=TRUE)

groups <- levels(as.factor(annotation_data$group))
colors <-  c("#999999","#000000","#111111")

annotations <- data.frame(
  txt_idat=annotation_data$idat,
  sampleName=annotation_data$sample,
  GROUP=mapvalues(annotation_data$group, from=groups, to=colors)
)

legends <- list(
  group=list(names=rev(groups), colors=rev(colors))
)
```

Selection of beta values

```
betaCriteria.table <- apply(betas,1,sd)
selected.cpgs <- sort(betaCriteria.table, decreasing=TRUE)[1:20000]
beta.table.filter <- betas[(rownames(betas) %in% names(selected.cpgs)),]
```

Clustering

Calculating distance

```
distance <-  dist(t(beta.table.filter),method='euclidian')
```

Cluster analysis

```
hr <- hcluster(beta.table.filter, nbproc=8)
hc <- hclust(distance, method='ward.D2')
```

Plotting

Creating the plot

The following command creates the plot with the help of the helper methods.

```
plotHeatmap('figure6a.pdf',
            act.hc = hc,
            act.hr = hr,
            act.betas = beta.table.filter,
            act.annotations = annotations,
            legends= legends)
```

Plotting helper methods

```
theme_none <-     theme(
  panel.grid.major = element_blank(),
  panel.grid.minor = element_blank(),
  panel.background = element_blank(),
  axis.title.x = element_blank(),
  axis.title.y = element_blank(),
  axis.text.x = element_blank(),
  axis.text.y = element_text(size=rel(1.2)),
  axis.ticks.y = element_blank(),
  axis.line = element_blank()
)


annotPlot <- function(annotationsClean, index) {
  colorCode <- annotationsClean[[index]]
  colName <- gsub("AND", "&",
                  gsub("X","X",
                       gsub("\\."," ",
                            gsub("_"," ",
                                 names(annotationsClean)[[index]]))))
  annotDF <- data.frame(
    x = 0:(length(colorCode)-1),
    y = 1
  )
  ggplot(annotDF, aes(xmin = x, xmax = x+1, ymin = 0, ymax = y) )+
    geom_rect(fill=colorCode)+
    geom_hline(aes(yintercept=0), size=0.5, color="#9C9C9C") +
    geom_hline(aes(yintercept=1), size=0.5, color="#9C9C9C") +
```

```r
    scale_x_continuous(limits=c(0, length(colorCode)), expand=c(0,0)) +
    scale_y_continuous(limits=c(-0.01,1.01), expand=c(0,0), breaks=c(0.5),
                       labels=c(colName)) +
    theme_none +
    theme(
      axis.text.y=element_text(size=rel(2), color="black"),
      legend.position="none",
      axis.ticks.x = element_blank(),
      plot.margin= unit(c(-0.2,0.5,-0.3,0.5),"lines"))
}

legendComplete <- function(legendsDF) {
  countLegends <- length(legendsDF)
  colorCode <- lapply(legendsDF, function(x) rev(x[["colors"]]))
  colName <- lapply(legendsDF, function(x) rev(x[["names"]]))
  title <-   gsub("XX","",gsub("\\."," ",gsub("_"," ",names(colName))))
  ySize <- sum(sapply(colName, function(x) length(x)))
  names <- c()
  colors <- c()
  for (i in 1:countLegends){
    names[length(names) +1 ] <- title[i]
    colors[length(colors) +1 ] <- "#FFFFFF"
    for( j in 1:length(colName[[i]])){
      names[length(names) +1 ] <- colName[[i]][[j]]
      colors[length(colors)+1] <- colorCode[[i]][[j]]
    }
  }
  names <- rev(names)
  colors <- rev(colors)
  annotDF <- data.frame(
    x = 1,
    y = 0:(length(names)-1)
  )
  ggplot(annotDF, aes(x=x+0.6, y=y+0.25, xmin = 0, xmax = x,
                      ymin = y + 0.1, ymax = y+0.9) )+
    geom_rect(fill=colors, linetype="solid",color="black")+
    geom_text(size=rel(6), hjust=0, vjust=0,  label=names) +
    scale_x_continuous(limit=c(0,10),expand=c(0,0)) +
    scale_y_continuous(limits=c(0, length(names)+0.5), expand=c(0,0)) +
    theme_none +
    theme(
      plot.title = element_text(size=rel(2.5)),
      axis.text.y = element_blank(),
      legend.position="none",
      axis.ticks.x = element_blank(),
      plot.margin= unit(c(0,0.5,-0.2,-0.5),"lines"))
}

getHeatmap <- function(act.hc, act.hr, act.betas, act.annotations, legends,
                       plotBetas=NULL, sampleNames=TRUE, act.width=25, act.height=25,
                       filename="tmpHeatmap"){

  dd.col <- as.dendrogram(act.hc)
```

```
col.ord <- order.dendrogram(dd.col)

dd.row <- as.dendrogram(act.hr)
row.ord <- order.dendrogram(dd.row)

xx <- act.betas[row.ord, col.ord]
xx_names <- attr(xx, "dimnames")

act.annotations <- act.annotations[match(xx_names[[2]],act.annotations$txt_idat),]
act.annotationsClean <- act.annotations[,!(colnames(act.annotations) %in%
                                             c("sampleName","txt_idat")),
                                        drop=FALSE]

df <- as.data.frame(t(xx))
colnames(df) <- xx_names[[1]]
df$samples <- act.annotations$sampleName
df$samples <- with(df, factor(samples, levels=samples))

mdf <- melt(df, id.vars="samples")

ddata_y <- dendro_data(dd.col)

act.annotPlots<- lapply(seq_along(act.annotationsClean),
                        function(x) annotPlot(act.annotationsClean, x))
act.legendPlots <- legendComplete(legends)

if(!is.null(plotBetas) && ncol(plotBetas)==ncol(act.betas)){
  plotBetas <- plotBetas[,match(xx_names[[2]],colnames(plotBetas))]
  plotBetas.df <- as.data.frame(t(plotBetas))
  plotBetas.df$nr <- 0:(ncol(plotBetas)-1)
  plotBetas.df$nr <- plotBetas.df$nr+0.5
  cpgNames <- colnames(plotBetas.df)
  for(i in 1:nrow(plotBetas)){
    actDF <- plotBetas.df[,c(i, ncol(plotBetas.df))]
    colnames(actDF) <- c("cpg","nr")
    plotBetasPlot <- ggplot(actDF, aes(nr, cpg)) + geom_point(size=1) +
      scale_x_continuous(limits=c(0, nrow(plotBetas.df)), expand=c(0,0)) +
      scale_y_continuous(limits=c(-0.05, 1.05), expand=c(0,0), breaks=c(0,0.5,1)) +
      theme_none +
      theme(  legend.position="none",
            axis.ticks.y = element_line(colour="#9C9C9C"),
            axis.ticks.x = element_blank(),
            panel.border = element_rect(fill=NA,color="darkgrey",
                                        size=0.5,linetype="solid"),
            plot.margin= unit(c(-0.1,0.5,-0.6,0.5),"lines"))
    act.annotPlots <- c(act.annotPlots, list(plotBetasPlot))
  }
}

if(!is.null(otherNumericVariables)){
  for (x in otherNumericVariables) {
    act.var <- x[match(xx_names[[2]],x$txt_idat),]
    act.var$sampleName <- as.character(act.var$sampleName)
```

```
    act.var$nr <- 0:(nrow(act.var)-1)
    act.var$nr <- act.var$nr+0.5
    act.var$bmi <- as.numeric(as.character(act.var$bmi))
    act.max <-  round(max(act.var$bmi, na.rm=TRUE))
    act.median <- as.numeric(round(median(act.var$bmi, na.rm=TRUE)))

    act.varPlot <- ggplot(act.var, aes(nr, bmi)) + geom_point(size=1) +
      scale_x_continuous(limits=c(0, nrow(act.var)), expand=c(0,0)) +
      scale_y_continuous(limits=c(-5, act.max+10), expand=c(0,0.1),
                         breaks=c(0,act.median,act.max)) +
      theme_none +
      theme(  legend.position="none",
            axis.ticks.y = element_line(colour="#9C9C9C"),
            axis.ticks.x = element_blank(),
            panel.border = element_rect(fill=NA,color="darkgrey",
                                        size=0.5,linetype="solid"),
            plot.margin= unit(c(-0.1,0.5,-0.6,0.5),"lines"))
    act.annotPlots <- c(act.annotPlots, list(act.varPlot))
  }
}

p1 <- ggplot(mdf, aes(samples, variable)) + geom_tile(aes(fill = value)) +
  scale_fill_gradient2(low = "blue", high = "red", midpoint=0.5) +
  scale_x_discrete( expand=c(0,0)) +
  scale_y_discrete(expand=c(0,0)) +
  theme_none +
  theme(axis.line=element_blank(),
        axis.text.x=element_blank(),
        axis.text.y=element_blank(),
        axis.ticks=element_blank(),
        axis.title.x=element_blank(),
        axis.title.y=element_blank(),
        legend.position="none",
        panel.background=element_blank(),
        panel.border=element_blank(),
        panel.grid.major=element_blank(),
        panel.grid.minor=element_blank(),
        plot.background=element_blank(),
        plot.margin=unit(c(0,0,-0.2,-0.2),"lines")) +  labs(x=NULL, y=NULL)

factor.png = 100

heatName <- paste("/tmp/",filename,".png",sep="")
heatName <- gsub("out/","",heatName)
png(heatName,res=300, width=act.width* factor.png,height=act.height*factor.png)
plot(p1)

dev.off()
p1.png<- readPNG(heatName)

# Create grobs to add to plot
my_g <-  rasterGrob(p1.png, interpolate=TRUE, width=1, height=1)
act.var <- data.frame(nr = (0:(ncol(act.betas))))
```

```
  act.var$var <- act.var$nr
  g <-   ggplot(act.var, aes(nr, var)) +
    scale_x_continuous(limits=c(0, act.width), expand=c(0,0)) +
    scale_y_continuous(limits=c(0, act.height), expand=c(0,0)) +
    annotation_custom(my_g,xmin=-Inf, xmax=Inf, ymin=-Inf, ymax= Inf)+
    theme_none  +
    theme(axis.text.y = element_blank(),
          axis.text.x = element_blank(),
          axis.ticks = element_blank(),
          plot.margin= unit(c(0,0,0,0),"lines"))
  # Dendrogram 1
  p2 <- ggplot(segment(ddata_y)) +
    geom_segment(aes(x=x, y=y, xend=xend, yend=yend)) +
    scale_x_continuous(limits=c(0.5, nrow(ddata_y$labels)+0.5), expand=c(0,0),
                       labels= df$samples, breaks=1:nrow(df)) +
    scale_y_continuous(expand=c(0,0)) +
    theme_none  +
    theme( axis.text.y = element_blank(),
          axis.ticks = element_blank(),
          axis.text.x = element_text(angle = 90, hjust = 0.5, size=rel(1)),
          plot.margin= unit(c(0.5,0.5,0.5,0.5),"lines"))

  if (!sampleNames) {
    p2 <-  p2 + theme( axis.text.x =element_blank(),
                      plot.margin= unit(c(0.5,0.5,-0.5,0.5),"lines"))
  }
  ggsave("/tmp/dendro.png",p2)
  completePlots <- c(list(p2),act.annotPlots,list(g))
  grobs <- list()
  widths <- list()
  for (i in 1:(length(completePlots))){
    grobs[[i]] <- ggplotGrob(completePlots[[i]])
    widths[[i]] <- grobs[[i]]$widths[1:5]
  }
  maxwidth <- do.call(grid::unit.pmax, widths)
  for (i in 1:length(grobs)){

    grobs[[i]]$widths[1:5] <- as.list(maxwidth)
  }
  legendGrob <- ggplotGrob(act.legendPlots)
  list(heatmap=grobs, legend=legendGrob)
}

plotHeatmap <- function(act.path=act.path, act.width=160, act.height=100, ...) {
  outPlots <- getHeatmap(act.width=act.width, act.height=act.height,...)
  pdf(act.path, width=act.width, height=act.height)
  heatmapAndAnnotations <-
    do.call("arrangeGrob",
            c(outPlots[["heatmap"]],
              list(nrow=length(outPlots[["heatmap"]]),ncol=1,
                   heights=c(0.2,rep(0.015,
                                     length(outPlots[["heatmap"]])-3),0.2,1))))
  legendPlots <-  arrangeGrob(outPlots[["legend"]],nrow=1)
```

```
grid.arrange(heatmapAndAnnotations, legendPlots, ncol=2, widths=c(1,0.3))
dev.off()
```
